# Anti-COVID drugs: repurposing existing drugs or search for new complex entities, strategies and perspectives

**DOI:** 10.4155/fmc-2020-0204

**Published:** 2020-07-23

**Authors:** Anna Pawełczyk, Lucjusz Zaprutko

**Affiliations:** ^1^Poznan University of Medical Sciences, Department of Organic Chemistry, Grunwaldzka 6, Poznań 60-780, Poland

**Keywords:** antiviral drugs, COVID-19, double hit effect, hybrid drugs, oleanolic acid, repurposing, SARS-CoV-2

## Abstract

At the end of 2019, a novel virus causing severe acute respiratory syndrome spread globally. There are currently no effective drugs targeting SARS-CoV-2. In this study, based on the analysis of numerous references and selected methods of computational chemistry, the strategy of integrative structural modification of small-molecules with antiviral activity into potential active complex molecules has been presented. Proposed molecules have been designed based on the structure of triterpene oleanolic acid and complemented by structures characteristic of selected anti-COVID therapy assisted drugs. Their pharmaceutical molecular parameters and the preliminary bioactivity were calculated and predicted. The results of the above analyses show that among the designed complex substances there are potential antiviral agents directed mainly on SARS-CoV-2.

For almost half a year now, we have been witnessing an uncontrolled spread of an epidemic, then reclassified as a pandemic, caused by the coronavirus known as SARS-CoV-2 [1,2]. The main initially observed symptoms of COVID-19 – the disease caused by SARS-CoV-2 – are fever, cough and shortness of breath. Over time, the National Health Organisation expanded the list of symptoms by adding muscle pain, general weakness and tiredness, and chills which may lead to convulsions [3]. In addition to these manifestations, various groups of scientists representing highly reputable research centers continue to report new symptoms tied to COVID-19. The US Centers of Disease Control and Prevention (CDC) have recently highlighted the widely observed prevalence of very specific manifestations relating to the loss of smell and taste [4,5], preceding by a few days the onset of the above-mentioned primary symptoms of infection. Also, there have been ample reports of strong headache [6,7] or purple lesions appearing on the hands and feet of COVID-19 patients, especially younger ones [8]. Recently, German researchers have announced that a decrease in albumin and antithrombin III levels in urine below 60% of the normal values is another early manifestation indicating a high risk of severe course of coronavirus infection [9]. Physically presenting symptoms and biochemical and immune markers are very numerous, but appropriate statistical validation is needed to evaluate their significance.

According to recent reports, many people who become infected with SARS-CoV-2 experience no symptoms. Consequently, it is difficult to determine the actual rate of infection in the general population. Among the individuals with confirmed infection or the presence of antibodies indicating past infection, there is a large group of patients presenting with only mild symptoms. On the other hand, in a percentage of patients SARS-CoV-2 may cause acute pneumonia with respiratory failure. In extremely severe cases, COVID-19 may lead to sepsis, septic shock and ultimately death. The estimated mortality rate due to COVID-19 covers a wide range, depending on the time of preparing the estimate and the geographical area to which the estimate relates. The mortality rate varies from approximately 2.5 to over 5%, and in some specific reference systems it may turn out to be significantly higher.

Currently, there are two methods used to control viral diseases. One of them involves the development of an appropriate vaccine targeting a specific strain of the virus, which, in the case of SARS-CoV-2, fortunately has not as yet shown a strong tendency to mutate. But in the last time more and more information about mutation possibility is published. The mutation rate in April 2020 was estimated at the level of about 30% and it varied depending on the region of the world [10]. The other authors [11] express this parameter as 1.05 × 10^-3^ to 1.26 × 10^-3^ substitution per site per year. The second method consists of using a drug which is a specific chemical substance inhibiting the growth and multiplication of the virus at any stage of its development. Potential drugs, in addition to high-molecular forms containing peptide or antibody units, include so-called ‘small-molecule chemicals’, characterized by the ability to interact with any of the key enzymes implicated in the development of this pathogen. High hopes are also being placed on the use of plasma from people who have recovered from COVID-19 and are capable of producing appropriate antibodies to attack the virus. These antibodies, following appropriate isolation process, can be incorporated into treatment as a specific therapeutic agent.

The purpose of this paper is to discuss chemical substances which have recently been selected as appropriate for experimental therapies – primarily for symptomatic treatment, though they were originally expected to mainly target the underlying cause. Most of these entities have already been used, with a greater or lesser degree of therapeutic success, as drugs with primarily antiviral activity. The group also includes antibacterial, antimalarial and antiparasitic agents, and other drugs. In this context, immense importance has been given to the so-called drug repurposing, which involves the identification of new areas of application and use for well-known and widely used chemical molecules. However, equally great significance should be attached to the search for completely novel molecules in the hope of finding at least one substance effectively controlling SARS-CoV-2. Among ongoing reports published on the Internet, there is ample information on companies searching through various databases to identify and propose a molecule not yet used in the treatment of viral diseases, but effectively combating the symptoms of COVID-19. Hundreds of thousands of chemical entities have already been analyzed in this manner, but no new structure has been proposed as yet, except for repurposed drugs. In pursuing the goal of this paper, based on the available literature and our own experience, we would like to propose a direction for the structural modifications of selected chemical compounds aimed at obtaining a product with possibly the greatest potential for the application against SARS-CoV-2.

## Repurposing existing drugs

This extremely topical issue has been selected as a research target by a number of interdisciplinary scientific teams. Saber-Ayad *et al*. [12] conducted a thorough up-to-date review of existing drugs with different targets and mechanisms of action, considered as candidates for repurposing for the treatment of COVID-19. In addition, potential side effects, threats and risks associated with the use of different substances were discussed. Unfortunately, the paper does not present any information on the chemical structure and the potential for its modification. Vanden Eynde [13] performed a brief review of clinical trials in two complementary papers published 1 month apart because of the unique dynamics of the situation both with respect to the number of conducted studies, their design and main focus.

Probably the most advanced studies aimed at the repurposing of drugs to obtain anti-COVID activity, complemented by an analysis of possibilities for successfully combining different substances, have been conducted by a team from Ohio, USA [14]. In their study, based on bioinformatics analysis and generated networks of functional drug–gene interactions, the authors identified 16 well-known but especially promising substances with different recognized primary indications and, in each case, with concurrent antiviral properties. They include irbesartan, toremifene, camphor, equilin, mesalazine, mercaptopurine, paroxetine, sirolimus, carvedilol, colchicine, dactinomycin, melatonin, quinacrine, eplerenon, emodin and oxymetholone. Furthermore, the authors suggested that a potentially significant therapeutic effect can be achieved by combining two drugs for concurrent administration. Such potential pairs include sirolimus with dactinomycin, mercaptopurine with melatonin and toremifene with emodin.

## Antiviral drugs

**Figure 1.**
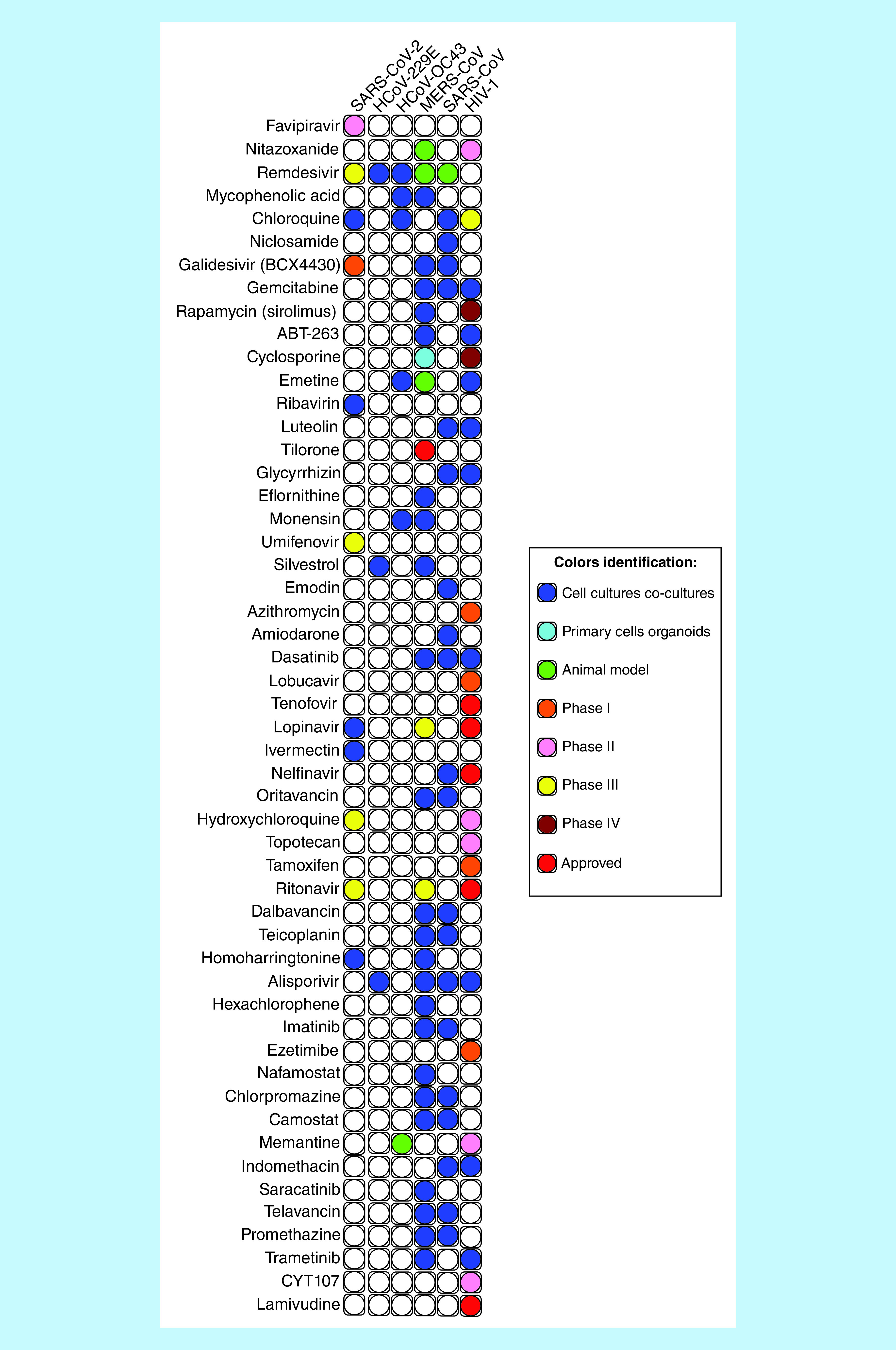
Advancement of research on selected drugs used against various types of viruses, and possibilities for their repurposing for SARS-CoV-2 therapy. The presented data are subject to current changes related to the introduction of individual drugs into experimental therapies.

Already in January 2020, Andersen *et al*. [15] reviewed the possibilities for repurposing known broad-spectrum antiviral drugs. An interactive diagram presented on the website at https://drugvirus.info/ [16] lists currently available antiviral substances in relation to different groups of viruses against which they can be used, taking into account the current stage of clinical trials. The diagram, together with its abbreviated version [15], already includes data on SARS-CoV-2. The list (Figure 1) shows that two drugs, lopinavir and arbidol, now in Phase IV studies, are the closest to being widely used in COVID-19 therapy. Remdesivir, ritonavir and hydroxychloroquine are currently undergoing Phase III studies. Among the 43 drugs listed, there are also four antibiotics which have been found to be effective against other coronavirus strains. They include monensin, oritavancin, dalbavancin and teicoplanin, as well as azithromycin. In addition to drugs acting against other viruses, other candidate drugs for the repurposing process discussed here include a number of antibacterial, antifungal, antiprotozoal and antiparasitic (also antimalarial) agents, in other words collectively termed antimicrobial drugs, as well as anthelmintics.

Two substances included in the above list, hydroxychloroquine and chloroquine, which have long been used as antimalarial agents, were among the first to be selected as drugs with a potential to fight SARS-CoV-2. However, it was quickly found that the drugs had to be used at significantly higher doses than in the traditional indications in order to observe an improvement in COVID-19 patients. Such treatment was shown to increase the severity of dangerous side effects, predominantly cardiac abnormalities seen on the electrocardiogram (ECG). After the initial euphoria, most medical centers are currently withdrawing from the prophylactic and therapeutic use of chloroquine following reports that the risk of adverse effects outweighs the benefits of treatment. Lentini *et al.* [17] proposed a concept to account for the lack of therapeutic success which attributed the observed adverse effects to the use of racemic chloroquine. Based on that claim, they hypothesized that one of the enantiomers of the substance, obtained in the process of resolution, might exhibit superior therapeutic properties. However, no results of such studies have been reported to date. A substance sharing some degree of structural likeness to chloroquine, with a similar heterocyclic system, is noscapine, currently used mainly for its antitussive effects. The application of noscapine in the treatment of SARS-CoV-2 has been suggested by Ebrahimi [18].

## Cardiovascular drugs

Another proposed group of candidate drugs for repurposing contains agents acting on the cardiovascular system selected from the group of angiotensin II receptor antagonists, the so-called sartans. Gurwitz [19] suggested that losartan or telmisartan might be suitable for this purpose, but soon afterward Rothlin *et al.* [20] argued that out of sartan medicines only telmisartan could be used with caution to obtain therapeutic benefits.

Rabby [21] reviewed the most up-to-date pharmacotherapeutic methods, and indicated the possibilities for grouping well-known, previously identified antiviral drugs in various combinations, complementing them with products adopted from the so-called Traditional Chinese Medicine. The conclusions presented in this study are of a very general nature, but an analysis of substances combined into mixtures reveals a potential for the use of anti-inflammatory substances and JAK inhibitors.

Following the identification of the structure of proteins playing a key role in the replication of SARS-CoV-2, for example M^pro^ (main protein), applying the strategy of structure-based virtual and high-throughput screening, a large team of Chinese scientists [22] analyzed a database of 10,000 compounds, selecting six substances with the highest potential inhibitory activity toward this protein. The group includes ebselen, disulfiram, tideglusib, carmofur, shikonin and an imidazole derivative designated as PX-12. The structure of the same protein was also used to demonstrate, by employing *in silico* methods, the promising properties of an andrographolide isolated from the herb of *Andrographis paniculata* [23]. A high degree of similarity between the proteins of SARS-CoV-2 and other SARS and MERS coronaviruses was reported in [24], suggesting a possibility of using the so-called small molecules to combat viral infections of this type. Based on the same observation, Li and De Clercq [25] also selected a few antiviral drugs (from a list containing more than 50 agents) that were successfully used during an early stage of spread of the COVID-19 pandemic. The most important substances proposed for repurposing in COVID-19 treatment, and not included as repurposing in Figure 1, are listed in Table 1.

**Table 1.** Existing therapeutic agents which have been considered as candidate drugs for the treatment of COVID-19.

Drug name	Chemical group	Already known indications	Ref.
Tenofovir	Nucleotide analog	Antiviral	[25]
Oseltamivir	Polisubstituted cyclohexene	Antiviral, selective neuraminidase inhibitor	[26]
Pyrazofurin	Nucleoside analog	Antiviral, antibiotic, anticancer	[21,25]
Disulfiram	Disulfide	To treat alcohol dependence	[25]
Irbesartan	Imidazolone derivative	Antihypertensive	[14]
Mercaptopurine	Purine derivative	Antimetabolite, antineoplastic	[14]
Mesalazine	Aminosalicylic acid	Anti-inflammatory	[14]
Toremifene	Stilbene derivative	Antineoplastic	[14]
Eplerenone	Synthetic steroid	Diuretic	[14]
Paroxetine	Piperidine derivative	Antidepressant	[14]
Dactinomycin	Macrolide antibiotic	Antineoplastic, antibiotic	[14]
Melatonin	Indole derivative	Hormone	[14]
Equilin	Steroid, estrone derivative	Estrogen	[14]
Quinacrine	Acridinederivative	Antimalarial	[14]
Carvedilol	Carbazole deriv. of amino-2-propanole	Nonselective beta-blocker	[14]
Colchicine	Alkaloid	Anti-inflammatory	[14]
Camphor	Monoterpene	Antipuritic, antiinfective	[14]
Oxymetholone	Steroid	Anabolic	[14]
Ebselen (PZ51)	Selenazolon	Anti-inflammatory, cytoprotective	[22]
Tideglusib	Tiadiazolodion	Anti-Alzheimer's disease	[22]
Carmofur (HCFU)	5-Fluorouracil derivative	Anticancer	[22]
Shikonin (Alkannin)	Naphtoquinone derivative	Natural red-brown dye	[22]
PX-12	Imidazodisulfide	Thioredoxin inhibitor, anticancer	[22]
Amoxicillin	Beta-lactam antibiotic	Antibiotic	[27]
Azithromycin	Macrolide antibiotic	Antibiotic	[27]
Glycyrrhizin	Triterpene saponin	Sweet substance, antiulcerous	[27–29]
Oleanolic acid	Triterpene		[29]
Ursolic acid	Triterpene		[29]
Hederagenin	Triterpene		[29]
Curcumin	Diarylheptanoid		[29]
Coriandrin	Furoisocoumarin		[29]
Apigenin	Flavonoid		[29]
Rosmarinic acid	Phenolic acids ester		[29]

In addition, currently, COVID-19 therapy introduces a number of drugs that do not act directly on the virus but alleviate the effects of its action on the human body. These include numerous drugs from α-blockers group (used in the treatment of prostate diseases and hypertension) that counteract the so-called cytokine storm activating factor Nrf2. Combinations of substances of natural origin with strong antioxidant activity are also used for this purpose [27].

## Drug candidates from traditional Chinese medicine

Many recent publications have also addressed the application of the Traditional Chinese Medicine for the prevention of SARS-CoV-2 infection and the treatment of COVID-19 [26,28,30,31]. This type of therapy is based predominantly on an extremely diverse range of plant-based substances, often derived from plants specific to this geographical region. These natural raw materials often comprise whole parts of plants containing triterpene substances, or triterpene compounds separated from them (Table 1) [26,30]. The most reported product of this type is the root of various species of liquorice (*Glycyrrhiza* sp.), as well as glycyrrhetic acid which is isolated from this raw material, and its glycoside glycyrrhizin [26,30]. The activity of these compounds against other types of coronaviruses was already reported in 2003 [32]. This area of study was intensely explored by Tolstikov *et al.* [33] who synthesized and investigated a number of glycyrrhizinic acid derivatives. These studies were considerably expanded by Kazakova *et al.* [34,35], who showed the activity of different modified triterpene compounds against various types of viruses including SARS, HIV and influenza. For other triterpene compounds commonly found in nature – such as oleanolic acid, ursolic acid, and hederagenin, the results of molecular docking to the SARS-CoV-2 M^pro^ protein have been reported [29], with very promising outcomes pointing to extensive possibilities for future applications, especially of the first two compounds, in the design of new drugs against the virus. Their binding affinities to the protein were comparable to saquinavir which was used as a positive control. A similarly high drug-likeness potential was also found by the authors [36] for mixture of ascorbic acid, curcumin and glycyrrhizic acid as well as for single curcumin, coriandrin, apigenin, and rosmarinic acid (Table 1) [29]. A team of researchers from Indonesia [37] also explored the possibility of using substances of natural origin as potential main protease inhibitors in the treatment of COVID-19. Applying Lipinski’s rule of five as the first step, and then docking selected natural substances to the protein, it was found that several flavonoids widely occurring in nature, as well as curcumin, were characterized by similar parameters to the existing antiviral drugs nelfinavir and lopinavir. These findings show that the natural materials listed above, as well as substances such as kaempferol, quercetin, luteolin, naringenin and coumarin, should stimulate the search for new effective antiviral agents. Similar conclusions were presented by the authors of [38] who, after analyzing a database of over 32,000 substances of plant origin, selected nine compounds that interacted particularly well with protease 3CLpro which is also characteristic of previously studied coronavirus varieties affecting the human body – SARS-CoV and MERS-CoV. The compounds also included rosmarinic acid derivatives, complex flavonoid compounds, and an indole derivative – amarantine. All the compounds were found to bind to the above-mentioned protein in a similar manner and with a similar strength as classic antiviral drugs which were used as comparators. Later papers [39,40], published on an almost daily basis in various scientific journals, also discuss the results of docking individual substances of natural origin to receptor proteins characteristic of SARS-CoV-2. Studies pinpoint polyphenolic substances as compounds with the highest capacity to bind to various receptor proteins previously confirmed as characteristic of coronaviruses, and potentially active against SARS-CoV-2. Study [40], evaluating PPAR-γ agonist substances found in food as potential modulators of the cytokine storm, highlights the importance of curcumin and capsaicin, among other compounds, within this line of research.

For the remainder of this paper, it is very important to consider the antiviral activity (also against coronaviruses) of triterpene compounds [41], primarily oleanolic acid [42], that was systematically reported in the literature. The ability of ursolic acid and oleanolic acid to bind to the key propathogenic M^pro^ protein present in the SARS-CoV-2 molecule, and the fact that the two triterpenes exhibit exceptionally good ADME parameters and meet Lipinski’s rule of five, have been extensively validated in the study by Kumar *et al.* [43]. This is the main premise for the new molecules which are being designed by us based on the structure of oleanolic acid as the basic skeleton and complemented by elements characteristic of anti-COVID drugs.

Currently, many new proteins present in the SARS-CoV-2 are known every day. A review of such major protein molecular targets has been described in [44]. Each of them may, in the future, be a separate molecular target for molecules designed and proposed in this paper.

## Perspective

Many of the papers cited here point to the high potential of pentacyclic triterpenes as substances with antiviral properties, also effective against SARS-CoV-2. Our experience with modifications of such compounds indicates a number of relationships between the structure and activity of the combinations thus obtained. It is on the basis of triterpene compounds that we have proposed the theory of ‘molecular consortia’ [45], which are combinations of various active elements designed to act jointly to achieve an effect anticipated for such a new entity either within a single cell or a cell assembly. The first compound of this type which we described was a combination of oleanolic acid and aspirin [46] displaying a far more potent anti-inflammatory effect than the individual constituents. At the same time, it was found that the newly formed compound had a completely different mechanism of anti-inflammatory action than each of its components. This observation became a starting point for the design and synthesis of a number of new derivatives combining triterpenes and non-steroidal anti-inflammatory drugs (NSAIDs) [47,48] or anticancer substances [49,50]. The activities and observations outlined above have served as a foundation for our attempt to design potential triterpene derivatives equipped with additional elements of known antiviral (and similar) drugs which are currently undergoing intensive studies to determine their suitability for repurposing in COVID-19 therapy.

The first assumption adopted in the process was selecting the triterpene structure of oleanolic acid as the basic skeleton. This skeleton has a certain advantage over the widely studied glycyrrhetinic acid because it lacks the ketone group at the C-11 position which is responsible for a range of side effects including the inhibition of 11-β-dehydroxysteroid dehydrogenase and, consequently, mineralocorticosteroid activity leading to pseudoaldosteronism. In addition, the oleanolic acid molecule has only three chemically active sites, two of which are used for the planned modifications, while the third one, the double bond at the Δ-(12,13) position, is sufficiently resistant chemically, so it does not hinder the formation of appropriate 3,28-derivatives, and furthermore does not determine the biological characteristics of the molecule.

The first of the sites subjected to modification in the structure of oleanolic acid is the carboxyl group at the C-28 position. It can naturally combine with sugars, but chemical modifications lead to the formation of practically two types of derivatives within the group: esters and amides, mostly monosubstituted or cyclic. A comparison of biological activity of these two types of derivatives provides clear evidence for the superiority of amides, particularly in terms of their anticancer, anti-inflammatory activities and against Alzheimer’s disease [51–53]. A number of amides of various triterpene acids have been discussed in the literature. Among those subjected to biological analysis, morpholide has been identified most commonly as a derivative demonstrating superior properties in a particular experiment [54], though the studied group of compounds also included amides bearing a dialkylaminoalkyl substituent [52,53]. However, there are as yet no literature reports on a triterpene acid amide having a substituent characteristic of chloroquine or hydroxychloroquine.

**Figure 2.**
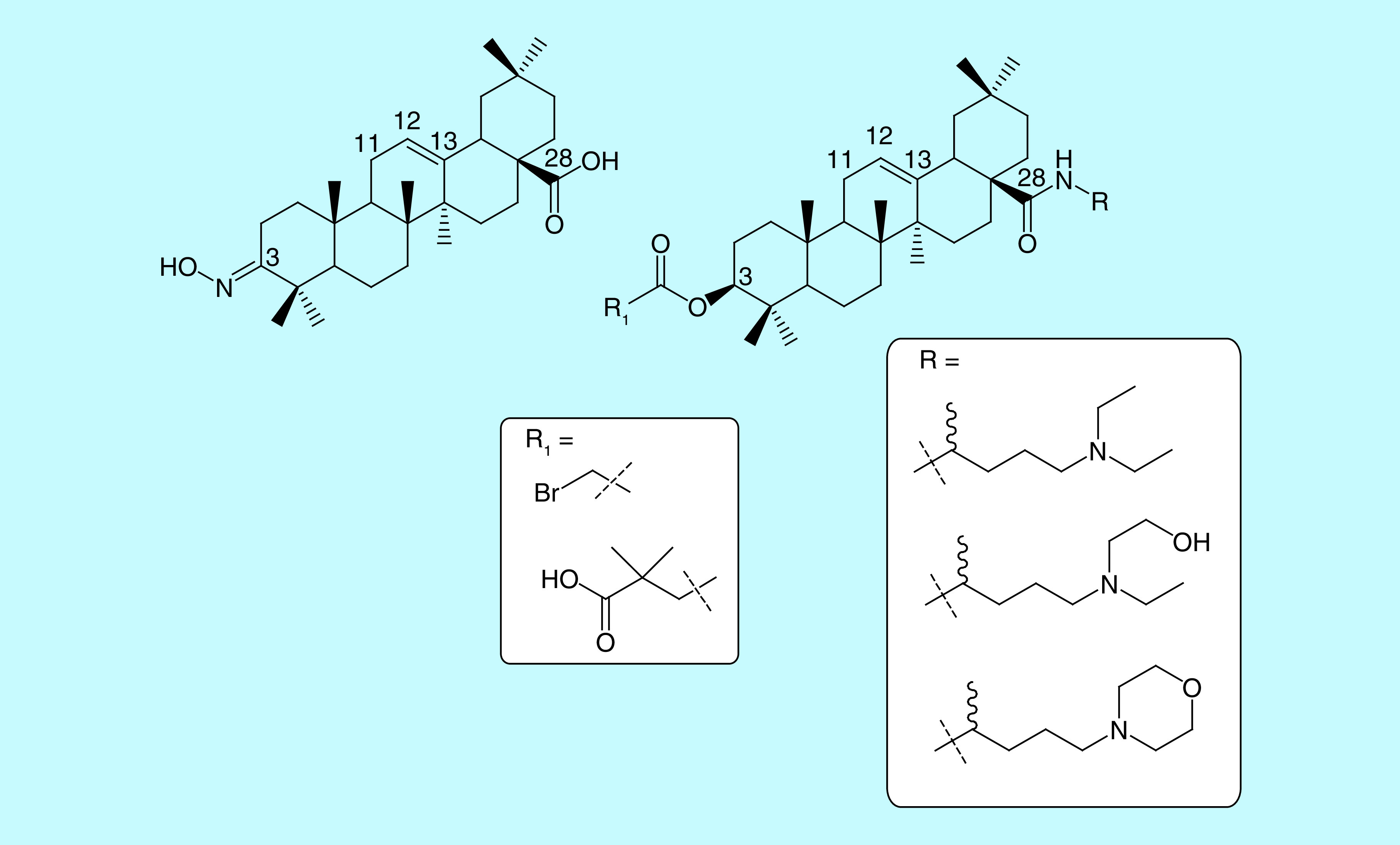
Selected derivatives of oleanolic acid modified at the C-3 and/or C-28 positions, showing a high level of biological activity (including antiviral effects).

This observation, coupled with the fact that chloroquine produces a range of side effects involving primarily the cardiovascular system (associated with the presence of a quinoline fragment in these molecules), became the basic assumption for the concept of incorporating into the oleanolic acid molecule an amide group substituted with a dialkylaminoalkyl chain present in chloroquine or hydroxychloroquine. This is to preserve the alkylamine arrangement, which is probably responsible for the antiviral activity and eliminate the fragment that accounts for the observed side effects. While developing this concept, we also proposed adding an amide monosubstituted with a 1-morpholine-4-pentyl group to the C-28 position of oleanolic acid. Such a substituent should combine the characteristics of the antiviral activity of chloroquine, reduce its side effects by introducing an oxygen atom at the end of the arrangement, and enhance the biological properties by incorporating that oxygen atom in a cyclic morpholine structure (Figure 2).

Another reactive site in the oleanolic acid molecule is the hydroxyl group at the C-3 position. In our previous studies, we have shown that the most beneficial cytotoxic effect is obtained after conversion of the group into the hydroxyimine group [55]. Such a group is also a carrier of high anti-inflammatory properties. Another group known from the literature [56], which is responsible for the activity of oleanolic acid derivatives against HIV-1, is a substituent in the form of a hemisuccinate ester, most advantageously 3,3-dimethylhemisuccinate (Figure 2).

**Figure 3.**
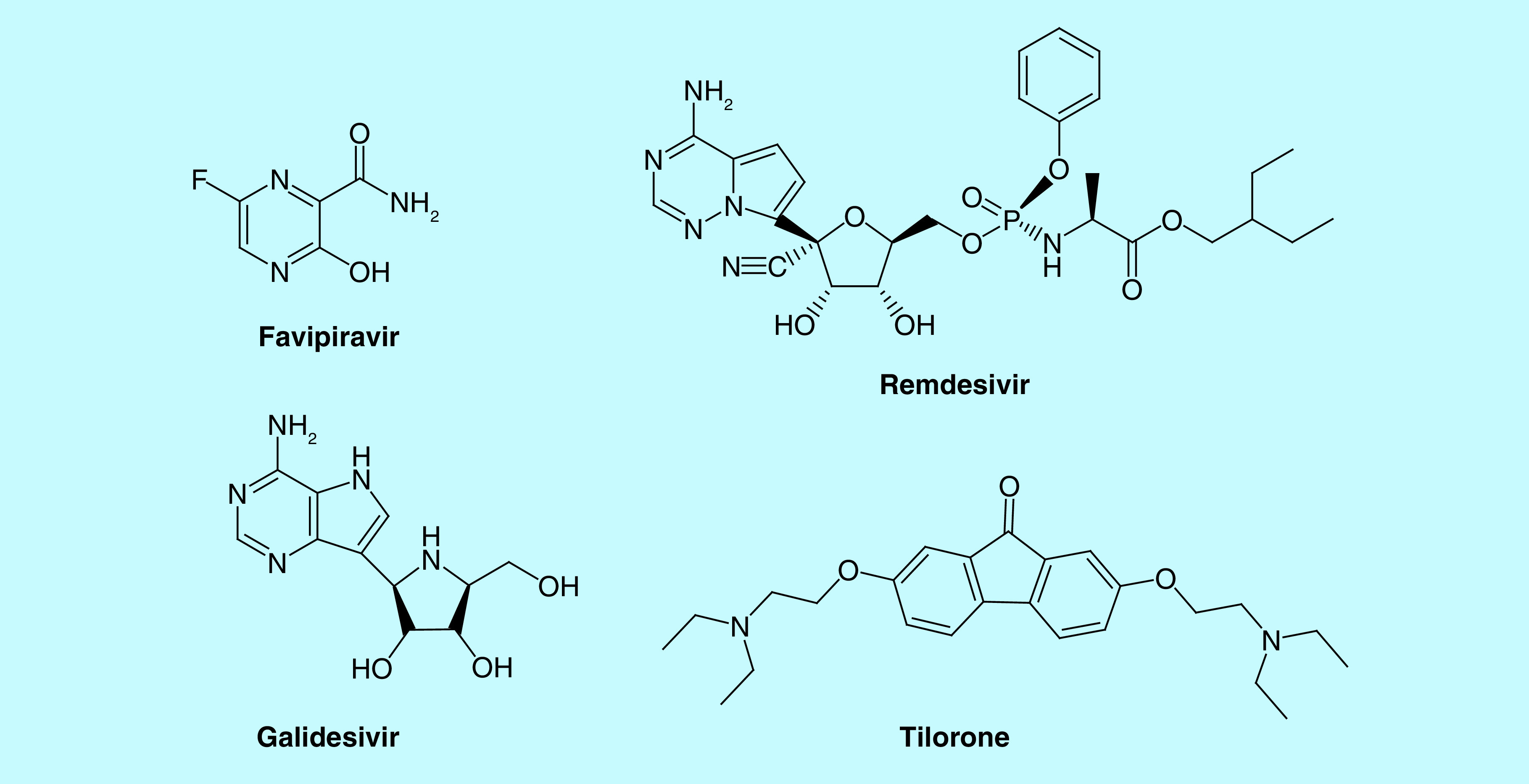
Structure of antiviral drugs proposed for the synthesis of ‘molecular consortia’ with oleanolic acid derivatives potentially exhibiting high activity against SARS-CoV-2.

We decided to use the above groups as linkers connecting the scaffold structure of oleanolic acid with antiviral drugs offering the greatest prospects for use in COVID-19 therapy identified in the process of repurposing known drugs. To this end, we performed an arbitrary selection of four drugs based on data included in [15,16], and numerous scientific reports published online on an ongoing basis. We decided to use the following agents: favipiravir, remdesivir, galidesivir and tilorone (Figure 3). Other typical antiviral drugs are highly structural complicated and therefore their conjugation with oleanane moiety can be low effective.

**Figure 4.**
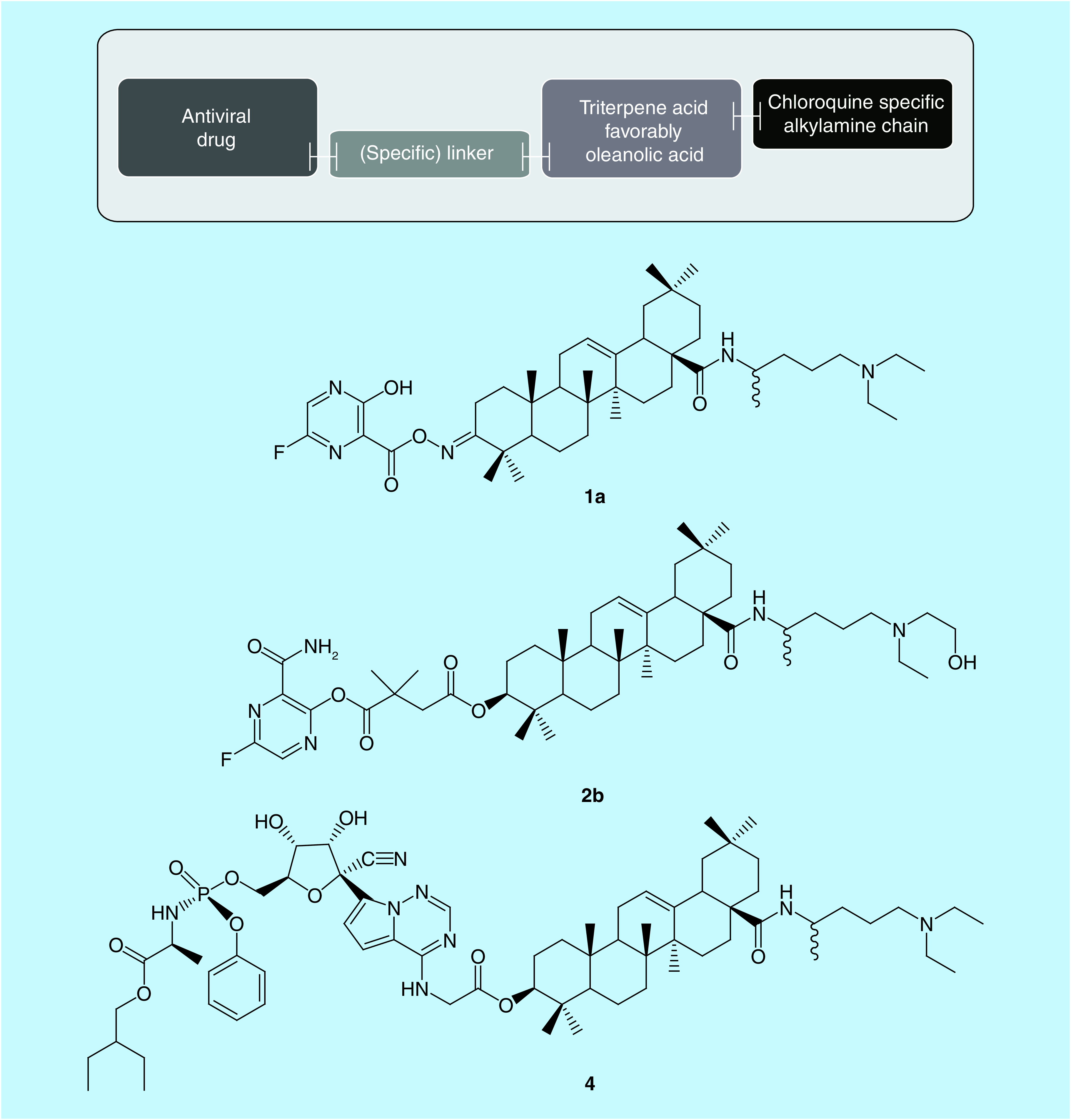
General block diagram and structure of new selected proposed anti-SARS-CoV-2 substances.

A combination of the above-mentioned elements results in proposed structures fulfilling the general criteria shown in the block diagram (Figure 4) and the detailed structures presented in Table 2.

**Table 2.** Simplified structures of proposed new antiviral drug oriented to coronavirus SARS-CoV-2.

Compound	Antiviral drug	Linker	Triterpene	Alkylamine moiety
**1a** **1b**	Favipiravir	Hydroxyimino group or dimethylsuccinate moiety	Oleanolamide	1-(diethylamino) pentan-4-yl
**2a** **2b**	Favipiravir	Hydroxyimino group or dimethylsuccinate moiety	Oleanolamide	*N*-ethyl-*N*-hydroxyethyl-(1-aminopentan)-4-yl
**3a** **3b**	Favipiravir	Hydroxyimino group or dimethylsuccinate moiety	Oleanolamide	1-morpholinpentan-4-yl
**4**	Remdesivir	Acetate moiety	Oleanolamide	1-(diethylamino) pentan-4-yl
**5**	Remdesivir	Acetate moiety	Oleanolamide	*N*-ethyl-*N*-hydroxyethyl-(1-aminopentan)-4-yl
**6**	Remdesivir	Acetate moiety	Oleanolamide	1-morpholinpentan-4-yl
**7**	Galidesivir	Dimethylsuccinate moiety	Oleanolamide	1-(diethylamino) pentan-4-yl
**8**	Galidesivir	Dimethylsuccinate moiety	Oleanolamide	*N*-ethyl-*N*-hydroxyethyl-(1-aminopentan)-4-yl
**9**	Galidesivir	Dimethylsuccinate moiety	Oleanolamide	1-morpholinpentan-4-yl
**10a** **10b**	Tilorone (oxime)	Acetate group or dimethylsuccinate moiety	Oleanolamide	1-(diethylamino) pentan-4-yl
**11a** **11b**	Tilorone (oxime)	Acetate group or dimethylsuccinate moiety	Oleanolamide	*N*-ethyl-*N*-hydroxyethyl-(1-aminopentan)-4-yl
**12a** **12b**	Tilorone (oxime)	Acetate group or dimethylsuccinate moiety	Oleanolamide	1-morpholinpentan-4-yl

Figure 4 also presents the structural formulas for the sample combinations listed in Table 2 with the highest expected antiviral activity (**1a**, **2b** and **4**), which as a result of combining the repositioning process and searching for new chemical molecules (useful in the therapy of SARS-CoV-2 virus infections).

### Main molecular parameters & preliminary bioactivity

For all the compounds listed in Table 2, a number of parameters mentioned in the Lipinski’s rule of five, characterizing these molecules as potential drugs were calculated. They were compared with relevant parameters of the constituent substances serving as conceptual precursors of the designed molecules. The calculations were performed using the Osiris Property Explorer [57] and Molinspiration Cheminformatics [58] software. The values characterizing different molecules obtained using both software packages were almost identical. Consequently, only the values acquired with one of them were included in the list, and it was indicated which software was the source of specific numerical data. The parameters describing the designed molecules obtained in this way are often considered too general to be adopted as a decisive criterion for their potential therapeutic use. However, a supplementary comparison of the designed structures with a database containing existing substances with established effects, in other words the application of the prediction of activity spectra for substances (PASS) method [59], adds validity to conclusions on the probability of achieving a specific desired effect with the designed structure. Applying the latter method, out of multiple possible directions of action, only those indicating antiviral activity of a given molecule were selected. It is widely recognized that Pa values over 0.7 signify a high probability of occurrence of a specific action, and if the value of this parameter exceeds 0.5, such action is regarded as possible and should be considered in further considerations.

All calculated parameters characterizing both components, including classic drugs (also antivirals), repurposed as anti-SARS-CoV-2 agents, and parameters describing newly designed molecules are listed in Table 3. Based on the data in the Table 3, conclusions can be drawn about the validity of assumptions on combining elements of small-molecule antiviral drugs into hybrids also referred to as molecular consortia. The concept, which has already been used with success, among others for anti-inflammatory substances, also proves its usability in the area of antiviral products.

**Table 3.** Main molecular parameters described by Lipinski's rule of five by Osiris Property Explorer^†^ and Molinspiration Cheminformatics^‡^ and predicted activity by prediction of activity spectra for substances method of proposed compounds.

Compound	MW^†^	clogP^†^	TPSA^†^	NHA^‡^	NHD^‡^	NRB^‡^	Solubility^†^	Drug likeness^†^	PASS P_a_ – antiviral			PASS – main activity
									General	Influenza	Hepatitis	
Oleanolic acid	456.0	6.06	57.53	3	2	1	-6.13	-1.78	0.493	0.836		chemopreventive hepatoprotectants
Chloroquine	319.0	4.01	28.16	3	1	8	-4.06	7.39	lack			antiprotozoal
Favipiravir	157.0	-0.74	89.10	5	3	1	-1.21	-1.99	0.321	0.434		antiallergic
Remdesivir	602.0	0.30	213.3	14	5	2	-4.99	-30.30	0.814		0.544	antiviral
Galidesivir	265.0	-2.21	140.3	8	7	2	-1.04	-0.1	0.571		0.373	purine nucleosidase inhibitor
Tilorone	410.0	4.06	42.01	5	0	12	-4.80	7.11	lack			CYP2 substrate
**1a**	749.0	8.78	117.0	9	2	11	-8.45	-3.39	0.329	0.668		NF-κB stimulant
**1b**	863.0	8.59	153.8	11	3	16	-9.17	5.63	0.333	0.654		NF-κB stimulant
**2a**	765.0	7.85	137.2	10	3	12	-7.94	2.9	0.324	0.665		NF-κB stimulant
**2b**	879.0	7.66	174.0	12	4	17	-8.66	5.22	0.330	0.667		NF-κB stimulant
**3a**	763.0	7.93	126.2	10	2	9	-7.91	0.88	0.323	0.608		NF-κB stimulant
**3b**	877.0	7.74	163.0	12	3	14	-8.63	3.16	0.329	0.582		NF-κB stimulant
**4**	1238.0	8.34	258.0	19	5	27	-11.72	-27.19	0.555		0.359	antineoplastic
**5**	1254.0	7.41	278.2	20	6	28	-11.26	-27.56	no data			no data
**6**	1252.0	7.50	267.2	20	5	25	-11.23	-29.52	no data			no data
**7**	971.0	7.37	205.0	14	7	17	-8.81	6.07	0.339			antineoplastic
**8**	987.0	6.44	225.2	15	8	18	-8.31	5.67	0.335			antineoplastic
**9**	985.0	6.53	214.2	15	7	15	-8.27	3.7	0.336			antineoplastic
**10a**	1061.0	12.6	105.1	11	1	25	-11.96	10.46	0.375			NF-κB stimulant and membrane integrity antagonist
**10b**	1131.0	13.94	122.2	12	1	27	-12.87	6.1	0.359			NF-κB stimulant and membrane integrity antagonist
**11a**	1077.0	11.67	125.4	12	2	26	-11.46	10.71	0.391			NF-κB stimulant and membrane integrity antagonist
**11b**	1147.0	13.01	142.4	13	2	28	-12.32	6.37	0.375			NF-κB stimulant and membrane integrity antagonist
**12a**	1075.0	11.75	114.4	12	1	23	-11.43	9.22	0.333			NF-κB stimulant and membrane integrity antagonist
**12b**	1145.0	13.01	131.4	13	1	25	-12.29	4.87	0.317			NF-κB stimulant and membrane integrity antagonist

^†^ Osiris Property Explorer data.

^‡^ Molinspiration Cheminformatics data.

clogP: Log of octanol/water partition coefficient; MW: Molecular weight; NHA: Number of hydrogen bond acceptors; NHD: Number of hydrogen bond donors; NRB: Number of rotatable bonds; Pa: Probability of activity predicted by the PASS method; PASS: Prediction of activity spectra for substances; TPSA: Topological polar surface area.

### Structure & potential bioactivity analysis

Among the newly designed compounds which are subject to analysis, two structural characteristics should be evaluated. One of them is the type of alkylamine substituent (derived from the chloroquine molecule or its analogs) attached via an amide bond at the C-28 position of the triterpene backbone. The other feature is the type of repurposed antiviral drug connected via a linker at the C-3 position in the oleanolic acid structure.

An evaluation of the former structural feature shows that the type of substituent terminating the aminoalkyl chain does not have a significant effect on the two key parameters of the molecule, in other words drug-likeness and P_a_ antiviral factor. For the compounds containing a diethylamine group, the level of drug-likeness varied from 10.46 to -27.19, and the P_a_ factor covered the range of 0.555–0.329. For the compounds with the *N*-ethanol-*N*-ethyl(amine) group, these values ranged from 10.71 to -27.56, and 0.391 to 0.324, respectively. In contrast, in the compounds with the morpholine substituent, the parameters assumed values from 9.22 to -29.52, and 0.336 to 0.317, respectively. Based on the distribution of different values within the above-mentioned ranges, the *N*-ethanol-*N*-ethyl(amine) group appears to demonstrate a slight advantage over the other two.

An evaluation of the other structural characteristic, in other words the type of antiviral drug repurposed for SARS-CoV-2, which is attached at the C-3 position of oleanolic acid, provides far more accurate insights into the planned directions of synthesis.

Based on the PASS method, potential antiviral activity at the P_a_ levels of 0.555 to 0.317 was demonstrated for all proposed structures. In this combination, a derivative containing remdesivir as an antiviral drug fragment appears particularly advantageous. However, using the activity prediction method, it was found that combinations of favipiravir with appropriately substituted oleanolic acid amide might exhibit activity against the influenza virus with a level of probability that is on average 1.5-times higher than that of the antiviral parent compound used for designing the molecular consortium-type structure. As for the designed compounds **1a**–**3a** and **1b**–**3b**, the P_a_ value defining their activity against the influenza virus varies from 0.668 to 0.582. This range, in conjunction with very favorable drug-likeness, varying on average from -3.39 to 5.63, is a good predictor of high suitability of these compounds in the treatment of viral diseases. An interesting conclusion arising from the analysis of the latter values is their variability depending on the type of linker connecting the triterpene to the antiviral drug, and hence also the use of a different favipiravir functional group in this combination. For the compounds **1a**–**3a** containing a hydroxyimine linker, the parameter of drug-likeness was between -3.39 and 2.90. For comparison, the compounds **1b**–**3b** with a linker in the form of dimethyl succinate group had a drug-likeness score of 3.16–5.63. These results show an advantage of the latter component of the designed molecules over the other components described here. This conclusion is also corroborated by the fact that a similar relationship was noted for the compounds **10a**–**12a** and **10b**–**12b**, which contained tilorone as a fragment showing antiviral activity. The fact that a similar effect associated with the presence of the dimethyl succinate linker is observed regardless of the drug molecule attached via this linker indicates that it is the structure of the linker, and not the way the drug binds to the linker, that contributes to improving drug-likeness properties.

The results of the above analyses show that among the designed complex structures of the molecular consortia-type with potential antiviral activity targeting mainly SARS-CoV-2, the highest therapeutic potential is noted in the compounds containing a molecule of flavipiravir in addition to the *N*-alkylaminoalkylamide fragment of oleanolic acid. With regard to the molecules with an added remdesivir fragment, the results are promising, however the excessively large molecular weights of the products make such outcomes uncertain, and potentially insufficient for drawing more general conclusions.

The proposed combinations **1b**–**3b**, as well as **1a**–**3a**, containing an element characteristic of chloroquine but without its undesirable side effects, and a fragment of favipiravir connected through the oleanolic acid structure, become multitarget drugs consistent with the concept of ‘double hit’ effect [60]. Owing to the presence of fragments of three drug substances with significant antiviral properties incorporated into one molecule that molecule will be active in at least two different phases of the coronavirus life cycle, as shown in the diagram in [61]. The dialkylaminoalkylamide fragment will be responsible for inhibiting viral replication at the stage of RNA release from the protein shell, while the favipiravir fragment will induce an inhibitory effect on the replication of viral genetic material.

## Conclusion

A great number of reports have recently been published to address the possibilities for repurposing antiviral drugs and developing new therapeutic agents and methods against SARS-CoV-2 [62,63]. All the concepts presented in these and many other available publications show that combining two ideas – repurposing and design of multitarget drugs – represents a valid approach. The practical application of these concepts and presented calculations will offer an opportunity to obtain new chemical entities which are highly likely to be useful in the fight with the COVID-19 pandemic.

## Future perspective

In order to launch an effective fight against SARS-CoV-2, for which there is no effective drug yet, two courses of action are possible. One is to develop a suitably specific vaccine, and the other is to find a chemical molecule that prevents infections and combats the virus.

Taking the second course of action, it can be noted that much importance in the fight with COVID-19 has been ascribed to the method of repurposing known drugs, especially those with an established activity against the influenza virus and used for the control of other diseases caused by microorganisms or protozoa. Drug repurposing is a method with a great potential which is still far too little used, and it may reveal itself as an extremely effective way of fighting diseases emerging in the future. Another well-proven, widely used and promising tool of modern medical chemistry is the search for completely new entities in the area of so-called small-molecule drugs. In addition, a currently fashionable, though not new, direction in the search for effective drugs involves taking advantage of the pharmacological potential of substances of natural origin.

The individual perspectives for the search of new nonbiological drugs belonging to the group of small-molecule chemical drugs against SARS-CoV-2, as addressed above, are discussed in a number of recent publications, for example by Akhtar [64], Amin and Jha [65]. In turn, this paper outlines a concept which ties together the above-mentioned individual trends, and proposes combining 2–3 elements with appropriate characteristics (subject to repurposing, natural origin, demonstrating biological activity and/or natural affinity for SARS-CoV-2 proteins) into one complex chemical molecule (a molecular consortium). In our view, the presented strategy is a promising direction with great prospective benefits in the area of searching for new pharmaceutical agents (especially multitargeted drugs) in the near future.

**Executive summary**

- There are currently no effective drugs targeting SARS-CoV-2.
- Topical methods used to control novel viral diseases is repurposing existing drugs or designing totally new entitled.
- Apart from antiviral drugs, in the search area are also drugs from other groups, for example cardiovascular, antibacterial, antimalarial, antiparasitic agents and drug candidates from traditional Chinese medicine.
- Triterpene derivatives for example oleanolic acid are agents with confirmed antiviral properties, also effective against SARS-CoV-2.
- Triterpene compounds, as the basic skeleton, equipped with additional elements of known antiviral (and similar) drugs are currently undergoing intensive studies to determine their suitability for repurposing in COVID-19 therapy.
- Favipiravir, remdesivir, galidesivir and tilorone as a antiviral drugs have been proposed for introduction into the oleanolic acid structure in C-3 position of the triterpene backbone by the hydroxyimino-, acetate- or dimethylsuccinate group as the linker.
- Alkylamine substituent (derived from the chloroquine molecule or its analogs) have been proposed for attachment via an amide bond at the C-28 position of the triterpene backbone.
- A combination of the above-mentioned three elements results in 18 designed complex structures with significant antiviral properties offering the greatest prospects for use in COVID-19 therapy.
- Based on the selected methods of computational chemistry the molecular parameters and the preliminary activity characterizing designed molecules as potential drugs were calculated and predicted.
- The results of the above analysis show that among the designed complex structures with potential antiviral activity targeting mainly SARS-CoV-2, the highest therapeutic potential is noted for the compounds containing a molecule of favipiravir in addition to the *N*-alkylaminoalkylamide fragment connected with oleanolic acid.
- Owing to the presence of fragments of three drug substances with significant antiviral properties incorporated into one molecule, the molecule will be active in at least two different phases of the coronavirus life cycle as a multitarged drugs.
